# Conserved Molecular Mechanisms Underlying Homeostasis of the Golgi Complex

**DOI:** 10.1155/2010/758230

**Published:** 2010-10-03

**Authors:** Cathal Wilson, Antonella Ragnini-Wilson

**Affiliations:** ^1^Department of Translational and Cellular Pharmacology, Consorzio Mario Negri Sud, Santa Maria Imbaro, 66030 Chieti, Italy; ^2^Department of Biology, Tor Vergata University of Rome, 00133 Rome, Italy

## Abstract

The Golgi complex performs a central function in the secretory pathway in the sorting and sequential processing of a large number of proteins destined for other endomembrane organelles, the plasma membrane, or secretion from the cell, in addition to lipid metabolism and signaling. The Golgi apparatus can be regarded as a self-organizing system that maintains a relatively stable morphofunctional organization in the face of an enormous flux of lipids and proteins. A large number of the molecular players that operate in these processes have been identified, their functions and interactions defined, but there is still debate about many aspects that regulate protein trafficking and, in particular, the maintenance of these highly dynamic structures and processes. Here, we consider how an evolutionarily conserved underlying mechanism based on retrograde trafficking that uses lipids, COPI, SNAREs, and tethers could maintain such a homeodynamic system.

## 1. Introduction

Despite the ancient origin of the Golgi and the differences in its structure across species, there is a striking conservation of a number of molecular machineries and principles that appear to operate in intra-Golgi trafficking. We sought to use these observations as a starting point from which to discuss how the maintenance of Golgi structure might be intrinsically related with the conservation of the basic molecular machineries that regulate intra-Golgi trafficking.

In most organisms the Golgi apparatus is composed of a series of flattened, membrane-bounded sacks (cisternae) arranged in a cis-to-trans fashion to form a stack. These stacks are laterally linked to form a ribbon-like membrane system in mammalian cells [1] but this ribbon-like structure does not link the Golgi stacks in plants and Drosophila [2, 3]. In the yeast *Saccharomyces cerevisiae* the Golgi compartments are not arranged as a stack at all but exist as separate scattered compartments in the cell [4, 5] while in some developmental stages of Drosophila no stacks are present [3]. Yet, the basic functions of the Golgi in transport and sorting appear to be conserved across species, so neither the stacked structure nor the ribbon can be considered as fundamental for the basic functions of the Golgi apparatus in transport and sorting of secretory cargo molecules.

In addition, it is possible to argue that ER-to-Golgi transport and the COPII complex, which is required for cargo selection and packaging at the ER [6], is not part of the self-organizing system per se. Many, but not all, Golgi-associated proteins recycle through the ER and are then reexported back to the Golgi in a continuous cycle [7–9]. Therefore ER-to-Golgi transport is required for constructing a Golgi, but only in the sense that it supplies some of the raw materials but is not part of the underlying mechanism that maintains the peculiar membrane organization of the Golgi. Therefore, here we do not consider COPII, which has been extensively reviewed elsewhere [10, 11], as part of the mechanism that maintains the homeostasis of the Golgi structure.

**Figure 1:**
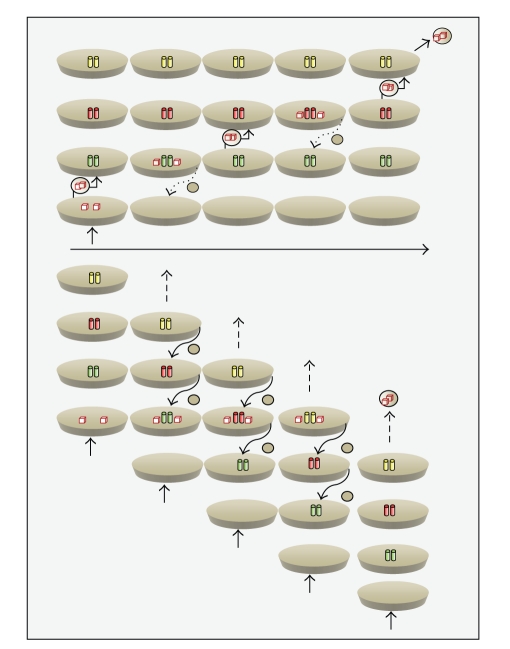
The vesicular transport model (top) versus the cisternal maturation model (bottom). In the vesicular transport model, cargo (red boxes) is transferred between stable Golgi compartments (coloured barrels) via vesicle carriers (circles with boxes) until it exits the Golgi (top right). Some proteins (e.g., SNAREs) and/or lipids could be returned via retrograde trafficking (curved downward-pointing arrows, unfilled circles). In the cisternal maturation model, the cargo can be considered to be stably located within a membrane compartment that changes identity via the retrograde trafficking (curved downward-pointing arrows, unfilled circles) of Golgi identity determinants (coloured barrels). As cargo leaves the Golgi in membrane carriers, the trans-Golgi is “consumed” (dashed vertical arrows) while new cisternae are forming by input from the ER (solid vertical arrows). Retrograde transport has a much greater role in maintaining an apparently stable system in the case of cisternal maturation. Under steady state conditions, both situations would appear to be the same. Large horizontal arrow: time of transport.

The Golgi complex can be considered a self-organizing system [12] where a dynamic equilibrium is maintained through multiple molecular interactions. Under steady state conditions the Golgi appears as a stable structure that was originally proposed to be a series of relatively stable cisternae while proteins destined for secretion or endomembrane compartments are transported via membrane carriers from the ER to the Golgi and from one cisterna to the next (the vesicular transport model). More recently the idea that the cisternae mature has gained favour [13]. In this model cargo is maintained within a cisterna that changes identity through the retrograde flow of compartmental identity proteins and lipids (the cisternal maturation model, Figure 1) [14]. Although both intra-Golgi trafficking and maintenance of Golgi structure are issues that are still far from being fully resolved [13], here we wish to discuss some possible mechanisms that, by depending on evolutionarily conserved intra-Golgi transport machineries, could underlie its peculiar membrane arrangement, concentrating in particular on interactions among lipids, SNAREs, tethers, and COPI-mediated retrograde transport that could generate the homeodynamic compartmental identity.

The Golgi compartments can be defined on one level by the presence of Golgi residents, such as glycosyltransferases, glycosidases, and sphingolipid synthesis enzymes that are organized in a graded fashion from cis to trans such that transported cargo is exposed in a sequential manner to the appropriate modification. This enzyme compartmentalization arrangement is conserved across species [18–20] so there must be conserved mechanisms that retain, or dynamically locate, the enzymes, all of which are integral membrane proteins, within a given location.

## 2. The Organizing Potential of LipidComposition

Phospholipids, cholesterol (ergosterol in yeast), and ceramide (the precursor for complex sphingolipids) are synthesized in the endoplasmic reticulum. The Golgi instead is the site of sphingolipid synthesis, primarily destined for export to the plasma membrane, in both mammals and yeast [21, 22]. Sterols are rapidly transported to other organelles so that the lipid composition of the ER is principally phospholipids while the PM is enriched in sphingolipids and cholesterol (ergosterol). The generation of a graded lipid composition across the Golgi is also influenced by the nonvesicular transfer of lipids between membranes via lipid transfer proteins such as CERT and FAPP2 [23, 24]. Additionally, fatty acid and cholesterol synthesis responding to glucose availability and sterol levels that depend on the SREBP-1c and SREBP-2 transcription factors, respectively, constantly maintain lipid levels at an optimal concentration [25]. 

Phosphoinositides (PIs), in particular PI(4)P, have an important role in recruiting proteins to the Golgi and in Golgi-to-PM transport. The PI(4)P levels depend on PI4KIII*β* kinase (Pik1 in yeast) for synthesis but their levels are also controlled by the phosphatase Sac1. This phosphatase is located in the ER under optimal growth conditions (growth factor stimulation in mammals or high glucose in yeast) as high PI(4)P levels are required for efficient transport. Removal of growth factors or glucose results in Sac1 translocation to the Golgi causing a decrease in PI(4)P levels and a shutdown of trafficking, effects that are fully reversible [26]. These observations point to a very dynamic equilibrium being monitored by the cell to maintain the correct lipid species and concentration across the secretory system. This has important implications for maintaining Golgi homeostasis as most Golgi-associated proteins are either transmembrane proteins or have affinity for a particular lipid species or membrane-lipid composition [27].

A good example of the membrane-protein interactions that may operate in maintaining Golgi homeostasis is the yeast Golgi-localised Vps74 protein (GOLPH3 in humans). PI(4)P is required for the correct localization of Vps74/GOLPH3, and both bind directly to PI(4)P. In pik1 mutants, the Golgi-localized Vps74/GOLPH3 is found in the cytosol. In the absence of the PI(4)P phosphatase Sac1, in which the PI(4)P levels are 10 times higher, Vps74/GOLPH3 is found in the ER and PM. Vps74 can bind glycosyltransferases as well as PI(4)P and interacts with multiple subunits of the COPI coat [28, 29]. Thus Vps74/GOLPH3 may act as a coincidence detector regulating retrograde trafficking and Golgi resident localization.

Protein retention in the Golgi or at the plasma membrane could be determined in part by differences in the thickness of the lipid bilayers conferred by the relative amount of cholesterol. Golgi membrane proteins tend to have a shorter transmembrane domain (TMD) compared to plasma membrane proteins so they are retained in the (relatively) cholesterol-poor thinner membranes of the Golgi while longer TMDs are retained in the thicker membranes of the PM [30–32]. However, the contribution of cholesterol to changes in membrane thickness has been contested since measurements of the bilayer thickness of ER, Golgi, basolateral, and apical membranes, contrary to expectations, do not match the cholesterol content of the membranes [33]. Rather it is proposed that mismatching of protein length and bilayer thickness results in changes in the properties of the bilayer leading to a high energy elastic deformation of the membrane. The bilayer lipids must deform to match the length of the TMD to avoid hydrophobic exposure. This could work in concert with lateral local changes in membrane thickness conferred by cholesterol-enriched domains, an effect that is reenforced by sphingolipids [34, 35]. Therefore, subdomains with lower sterol/sphingolipid content within the bilayer could sort proteins such that Golgi residents are excluded from those domains enriched in sterols/sphingolipids that contain forward moving cargo destined for the plasma membrane.

While the transmembrane domain of Golgi residents such as galactosyltransferases provides a dominant localization signal [36], the lumenal domain and cytoplasmic tail and the ability to form oligomers that depends on differences in pH across the Golgi are also important determinants [36–38]. Oligomerization could act as a mechanism for separating the Golgi residents, possibly via different lipid subdomains of the membrane, from cargo that are being transported in an anterograde manner.

COPI vesicles appear to contain less sphingolipids and cholesterol than their parental Golgi membranes [39], which might also explain why they exhibit a clearer interleaflet space than the membranes from which they bud [40]. It is possible that COPI vesicles function in the dynamic redistribution of lipids to maintain Golgi structure and/or that the lipid partitioning segregates Golgi residents into COPI vesicles for retrograde trafficking.

## 3. COPI-Mediated Retrograde Trafficking

The vesicular transport model envisages Golgi cisternae as stable structures where vesicles carrying cargo molecules in the anterograde direction are generated while COPI-mediated retrograde vesicles return “escaped” proteins to the endoplasmic reticulum (Figure 1). The cisternal maturation model, where each cisterna matures from early to late retaining the secretory cargo proteins but losing the Golgi resident proteins in a retrograde manner to the upcoming cisterna that then acquires the identity of the cisterna that went before it, requires a much greater role for COPI (Figure 1). Strong support for the cisternal maturation model came from the visualization of Golgi resident proteins that showed a dynamic transition from cis to trans compartmental identity in living yeast cells [41, 42].

Despite many studies, the role of COPI in intra-Golgi transport and Golgi maintenance is still a subject of debate and one of the major unresolved issues in providing a comprehensive explanation of Golgi function [13]. A number of studies have presented evidence for different populations of COPI vesicles with different protein compositions enriched in Golgi resident proteins but excluding anterograde cargo molecules [43–47]. However, others have contested the presence of Golgi enzymes in COPI vesicles [48, 49]. Using EM tomography, the Golgi enzymes were found to be enriched in perforated zones at the rims of the Golgi cisternae but to be excluded from COPI vesicles. Further, when cargo transport through the Golgi is blocked there appears to be an accumulation of peri-Golgi COPI vesicles while activation of transport leads to a decrease in these vesicles and the formation of tubular connections [50, 51].

Instead of requiring COPI vesicles for intra-Golgi retrograde transport, a mechanism for the retrograde transport of the Golgi residents could be via the development of intercisternal tubular connections by the action of phospholipase A_2_ *α* [51, 52]. This requires COPI-mediated recruitment of fusion machinery to generate COPI buds that, in the absence of fissioning machinery, would then result in tubulation [52]. In accordance with this, a lysophosphatidic acid-specific acyltransferase, LPAAT3, negatively regulates tubule formation and is important for Golgi structure [53]. Golgi tubules that can be induced to form at low temperature contain Gos28, GS15, Rab6, and glycosylation enzymes, but exclude Sec22, membrin, Rab1, and Rab2 that instead mediate ER-to-Golgi traffic, suggestive of intra-Golgi trafficking tubules [54]. 

One possibility is that intercisternal tubule and COPI vesicle formation are mechanistically related, the former mediating retrograde intra-Golgi transport via a COPI-dependent mechanism, but in the absence of fission [52], while the latter mediates Golgi-to-ER retrograde transport via COPI vesicles. COPI was reported to be concentrated at the cis-Golgi [55] and most reports on COPI-mediated trafficking refer to a cis-Golgi-to-ER retrograde pathway. However, COPI-isoforms are localized across the Golgi stack [56]. Retrograde transport via COPI vesicles, rather than returning “escaped” proteins to the ER, might act in regulating the dynamics of the ERGIC while retrograde transport between the Golgi cisternae could be instead mediated by tubule formation in the absence of scission, possibly involving PLA_2_ and phospholipase D [52, 57].

## 4. Arf1 GTPase: GEFs and GAPs

As for other small GTPases, Arf1 undergoes a GDP-GTP cycle that is regulated by guanine nucleotide exchange factors (GEFs) and GTPase activating proteins (GAPs) leading to a rapid on-off cycle with Golgi membranes [58]. It appears to be present across the Golgi stack and has multiple functions in recruiting functional determinants to the various compartments. The recruitment of Arf1 to different regions of the Golgi could depend on the differential localization/properties of the GEFs and GAPs [59, 60]. Arf1 plays a major role in cargo sorting and transport through the recruitment of multiple effectors and lipid modifying enzymes [61]. The role for Arf1 in maintaining Golgi structure, as apposed to sorting and trafficking, could be through its control of COPI vesicle formation. Inhibition of Arf1 activation by BFA, which leads to a nonproductive complex between Arf1-GDP and ArfGEFs, causes rapid disassembly of the Golgi and dispersal of many of the Golgi associated proteins to the ER or the cytosol [62]. A recent report showed that a highly specific inhibitor of the ArfGEF GBF1 caused a dissociation of COPI vesicle coats from Golgi membranes and Golgi disassembly pointing out its importance for the structural integrity of the Golgi [63]. Although Arf1 is important for these processes, it can be argued that it is the rate of activation/inactivation, mediated by GEFs and GAPs that are differentially located in the Golgi, and thus the rate of budding that is the true regulator in maintaining Golgi structure. These rate-determining interactions may be influenced by the lipid and phosphoinositide composition in the vicinity that might regulate their interaction [64, 65] while the lipid composition could also determine the recruitment of Arfs, GEFs, and GAPs and in turn be regulated by Arf1 in a positive feedback loop [63]. 

ArfGAPs have been shown to interact with different SNARE proteins. In yeast, the Glo3 and Gcs1 ArfGAPs recruit diverse SNAREs [66–68] and the mammalian ArfGAP Hrb binds the SNARE VAMP7 for sorting into recycling endosomal vesicles [69]. Coordination of Arf1 and coatomer recruitment by ArfGEFs and ArfGAPs together with SNARE binding could provide a mechanism for the regulated recycling of these SNAREs in COPI vesicles. SNARE proteins such as Gos28, Sed5, and membrin can be incorporated into and enriched in COPI vesicles with respect to cisternae [45, 47, 70–72].

## 5. Rab Proteins

The use of bacterial toxins has been informative in identifying a multitude of retrograde pathways since they exploit retrograde transport pathways from the plasma membrane to the ER and can resolve the interdependence of the anterograde/retrograde pathways. In particular, these studies have highlighted the role of the small GTPase Rab6 in retrograde transport. While toxins such as cholera toxin have a KDEL-like sequence and depend on COPI for delivery to the ER [73], others, such as Shiga toxin, are COPI independent but rely on Rab6, a pathway that may be also used for the recycling of glycosyltransferases [74, 75]. Yet another toxin, SubAB, depends on a COG-Rab6-COPI retrograde pathway [76]. The localization of Rab6 to medial/trans-Golgi cisternae is consistent with a role in retrograde intra-Golgi trafficking [77]. The interaction of Rab6 with the COG complex (see what follows) may be important for retrograde intra-Golgi trafficking and Golgi homeostasis [78] and these findings are supported by studies in S. cerevisiae of Ypt6p, the homologue of Rab6. Ypt6p, the only Rab6-like molecule in S. cerevisiae, interacts genetically with COG [79, 80] and detailed analyses of Ypt6 mutants show a defect in Golgi glycosyltransferase function/localization that is most probably due to defects in intra-Golgi retrograde transport [79].

## 6. Tethers in Retrograde Transport

The golgin family of proteins and tethering complexes has a role in the structural organization of the Golgi and in trafficking. A summary of golgins in different organisms (Table 1 and references therein) shows that a large number of them are not present in yeast or plants, suggesting that they play a role in Golgi ribbon formation in mammals [81]. The Golgi in Drosophila cells do not show ribbon formation but two stacks are tightly apposed that may represent a “minimal” ribbon that could explain the presence of these golgins [3]. 

Within the present context, the COG (Conserved Oligomeric Golgi) complex appears to be of particular relevance for retrograde intra-Golgi trafficking. This highly conserved peripheral membrane protein complex is proposed to act as a retrograde vesicle-tethering factor in intra-Golgi trafficking [15]. Downregulation of COG function in mammals and yeast results in the mislocalization of resident Golgi glycosyltransferases/glycosidases [82–84] and defects in the recycling of Golgi proteins [85]. COG subunits show genetic and physical interactions with intra-Golgi SNAREs and the COPI coat [86] and can bind to the t-SNARE Syntaxin5a/Sed5p thus enhancing the stability of intra-Golgi SNARE complexes [87]. Immunogold electron microscopy showed that the COG1 subunit is localized across the Golgi stack on or close to the tips and rims of the Golgi's cisternae and in some cases on COPI containing vesicles [88].

## 7. SNAREs as Generators of Compartments

Soluble N-ethylmaleimide-sensitive factor attachment protein receptor (SNARE) proteins are essential for the fusion of transport vesicles, or a donor membrane compartment, with an acceptor membrane. A complex of SNARE proteins that is localized in opposing membranes drives membrane fusion. The four SNARE proteins that contribute to the formation of the complex direct different trafficking steps at different locations in the cell. By and large, the combinations of the different SNARE proteins at different locations within the main intracellular trafficking pathways are conserved between organisms [89]. Interestingly, computer simulation modeling has suggested that this differential localization of the SNAREs could contribute to the generation and maintenance of stable nonidentical Golgi compartments. A differential affinity of a coat protein for one set of SNAREs over another could lead to the concentration of these SNAREs and this, together with the selective fusion with their cognate SNAREs, was sufficient not only to generate non-identical Golgi compartments de novo but also to maintain the steady state system of non-identical Golgi compartments [90, 91]. As mentioned above, this could be controlled via COPI vesicles and/or tubular connections.

## 8. Conclusions

**Figure 2:**
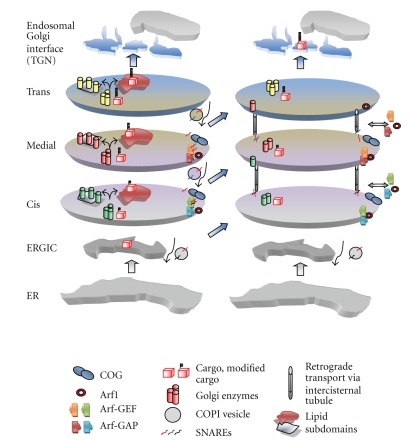
A model for maintaining Golgi structure during cisternal maturation by retrograde transport. Incoming cargo is modified by Golgi enzymes and then lipid partitioning separates cargo from enzymes (divergent arrows). Intra-Golgi transport could be controlled via COPI vesicles that regulate the composition and concentration of SNARE proteins and lipids in the cisternae. During anterograde transport the cargo molecules are maintained within a cisterna that changes identity by the retrograde recycling of SNAREs, Golgi resident proteins, and lipids via intercisternal tubules, with the COG complex acting as a tethering factor. Arf1 is present across the Golgi and regulates COPI vesicle formation but distinct domains are conferred by the ArfGEFs and ArfGAPs, all of which associate and dissociate rapidly from the Golgi membranes according to the changing lipid/protein identity (see text for details).

The Golgi apparatus shows characteristics consistent with it being a self-organizing system. Such a system relies on multiple interdependent interactions to maintain it in a homeodynamic state. In relatively simple systems, such as the cytoskeleton, it has been possible to describe the factors that regulate the self-organization [12]. However, in a system as complicated as the Golgi it is difficult to arrive at a simple underlying molecular mechanism that is responsible for its maintenance. However, a number of factors that have been described in relation to the functioning of the Golgi complex can be considered as operating together to generate and maintain this system. The mechanisms described above are conserved across species and are therefore applicable in describing the basic functioning of the Golgi (Table 2). We speculate that a combination of lipid partitioning, SNAREs, tethers, and retrograde trafficking that relies on COPI, could be sufficient to generate a compartmental system as seen in the Golgi (Figure 2). Lipid input from the ER and endosomal compartments and from the nonvesicular transfer of lipids between membranes via lipid transfer proteins could set up the basic membrane platform that recruits various lipid binding proteins that then organize the whole into a self-regulating homeostatic system by counterbalancing forward transport with the retrograde trafficking of proteins and lipids to generate differential protein and lipid composition across the Golgi cisternae. The concentration gradient of different lipids across the Golgi could determine the localization of Golgi residents that define the identity of the compartments. Further, this lipid sorting may be an important factor not only in regulating Golgi structure but also in providing a driving force for intra-Golgi cargo segregation and transport [92].

## Figures and Tables

**Table 1:** Golgins and tethers in Golgi structure and maintenance. Potential homologues in mammals, *Saccharomyces cerevisiae*, *Arabidopsis thaliana,* and *Drosophila melanogaster*. For a detailed description see [2, 3, 15]. Others not reported in these reviews are from [16, 17] or were searched for directly by BLAST searches at http://flybase.org/; http://www.arabidopsis.org/; http://www.yeastgenome.org/. *Trs65, Trs85 subunits of TRAPPII present only in fungi.

	Mammal	Yeast	Arabidopsis	Drosophila
p115	+	+	+	+
GM130	+	+	−	+
Golgin245	+	+	+	+
GMAP210	+	+	+	+
CASP	+	+	+	−
GRASP65	+	+	−	+
Golgin84	+	−	+	+
TMF	+	+	+	+
GCC88	+	−	−	+
GCC185	+	−	−	+
GCP60	+	−	−	+
Giantin	+	−	−	−
Golgin45	+	−	−	+
Golgin97	+	−	−	+
GRASP55	+	−	−	+
Golgin160	+	−	−	−
TRAPPI,II*	+	+	+	+
COG1-8	+	+	+	+

**Table 2:** Evolutionarily conserved molecules, machineries, and principles involved in intra-Golgi transport that could underlie the homeostasis of the Golgi complex.

Mammals	Yeast	Comment
Lipid metabolism Cholesterol/sphingolipids	Lipid metabolism Ergosterol/sphingolipids	Protein organization due to affinity for lipid subdomains

PI4KIII*β*	Pik1	Same regulatory mechanism underlying PI(4)P metabolism
Sac1	Sac1	

GOLPH3	VPS74	Coincidence detector for Golgi resident localisation
PI(4)P	PI(4)P	
(COPI)	COPI	

COPI	COPI	Different isoforms distributed over the Golgi

ArfGAPs	ArfGAPs	Different isoforms distributed over the Golgi
ArfGEFs	ArfGEFs	

Arf1	Arf1	Distributed over the Golgi

GOG1-4, GOG5-8	GOG1-4, GOG5-8	Two conserved COG subcomplexes

Rab6	Ypt6	Small GTPase governing retrograde transport

SNAREs	SNAREs	Same basic distribution in the Golgi

## Abbreviations

BFA: Brefeldin A
EM: Electron microscopy
ER: Endoplasmic reticulum
GAP: GTPase-activating protein
GEF: Guanine nucleotide-exchange factor
PM: Plasma membrane
SNARE: Soluble N-ethylmaleimide-sensitive factor attachment protein receptor
TMD: Transmembrane domain.
